# Psychological assessment during and after the COVID-19 pandemic

**DOI:** 10.4102/ajopa.v3i0.74

**Published:** 2021-09-02

**Authors:** Justin O. August, Solomon Mashegoane

**Affiliations:** 1 Department of Psychology, Faculty of Health Sciences, Nelson Mandela University, Port Elizabeth, South Africa; 2 Department of Psychology, Faculty of Humanities, University of Limpopo, Polokwane, South Africa

The coronavirus disease 2019 (COVID-19) has become a global pandemic, with increasing numbers of infected patients being reported daily. At the beginning of July 2021, the African continent had recorded a total of nearly 5.7 million COVID-19 cases and just over 147 000 deaths, whilst only just under 1.1% individuals have been fully vaccinated (Africa CDC COVID-19 Brief, 2021). Countries across the globe, including those on the African continent, have imposed restrictions on movement and social distancing, with most of them implementing some form of lockdown. The psychological, social and economic effects of the pandemic are unprecedented, most certainly like nothing this generation has ever experienced. The current COVID-19 climate has necessitated shifts in all aspects of human physical interaction, with remote working becoming the norm and teleservices for everything from healthcare to shopping taking prominence. The effects on marginalised communities remain a core concern (UN Committee on Economic, Social & Cultural Rights [UNCESCR], 2020), with the pandemic exacerbating structural inequalities and exposing the stark socio-economic realities that have often remained hidden in a pre-COVID-19 era.

Psychology and psychological assessment in particular had to re-examine its processes. The majority of assessment in Africa is person to person. Assessment practitioners in the African context already face challenges of lack of appropriate test material, language difficulties and constraints with regard to cross-cultural applicability (Mpofu & Nyanungo, 1998; Mpofu, Peltzer, Shumba, Serpell, & Mogaji, 2005; Laher & Cockcroft, 2013). Online assessments are atypical. In countries outside of the African continent, tele-assessment approaches are deployed during the emergency lockdown to continue psychological assessments, thus minimising face-to-face contact (British Psychological Society [BPS], 2021; Farmer et al., 2020; Health Professions Council of South Africa [HPCSA], 2020; Hewitt, Rodgin, Loring, Pritchard, & Jacobson, 2020). Across Africa, very few measures can be utilised via a tele-assessment medium, and even if they could, a large percentage of the population is at a disadvantage through this practice. Technological inaccessibility is a reality across the continent, particularly amongst the most marginalised populations (Mahler, Montes, & Locke, 2019).

The inequalities faced by the marginalised populations in society were always prevalent in assessment (Laher, Serpell, Ntinda, & Chireshe, in press; Oppong, Oppong Asante, & Adote Anum, in press), but have been exacerbated manifold during the pandemic. The teaching of psychological assessment at universities and training sites has also been a cause of concern. Guidelines from African organisations such as the HPCSA and international guidelines from the BPS have been generic, presenting overarching factors that practitioners should consider but input on the efficacy of these guidelines is still forthcoming. The practice of psychological testing and assessment requires rethinking, reconstructing and critical engagement (Hewitt et al., 2020). The articles in this special section explore various ways in which psychological assessment can be conducted during COVID-19 traversing a number of contexts from corporate organisations through to higher education in Africa.

Dowdeswell and Kriek (2021) explore the perceptions of 41 cross-industry clients for COVID-19 and post-COVID-19 human resources practices. One of the most interesting observations made by the authors is that the private sector is already on solid ground when it comes to online psychological assessment during the COVID-19 pandemic. Seemingly, the pandemic did not impose as harsh a need for transition in the sector as online testing was already being used in industry. They discuss the use of unproctored internet testing (UIT) and virtual or video interviewing technologies and the role of assessment in retrenchment and restructuring applications in industry. Of note is the argument around access to technology and the role of mobile devices in this process providing some equitable access even if far from ideal. Dowdeswell and Kriek predict that the post-COVID-19 work environment will take advantage of existing technology and maintain the momentum of digitalisation, suggesting that the pandemic and lockdown have offered opportunities for re-examining the traditional mode of assessment practices.

Munnik, Smith, Adams Tucker and Human’s (2021) article highlights how a South African institution responded to the constraints imposed by the emergency lockdown to the teaching and training of psychological assessment to masters students in a clinical psychology programme. Munnik et al. found that teaching and training under emergency lockdown conditions necessitated reprioritisation. The reconceptualised view of the teaching process meant that training in psychometrics can be seen as processual, involving varied stakeholders and settings. The authors discuss the use of multiple pedagogies in an attempt to incorporate theoretical and practical training online reflecting on the efficacy of these going forward.

Wigdorowitz, Rajab, Hassem and Titi (2021) invite test users to explore the complex challenges brought about by testing under conditions of online psychological assessment where physical contact is impossible. Cognitive testing is challenging because unlike personality testing, requisite testing conditions require some form of direct observation to supervise the process, control time and set up context. Wigdorowitz et al. considered all aspects of assessment in the private sector, including the added burden of shifting expenses to the test taker and the integrity of the testing process itself. For test users in academia, the incentives (e.g. employment requirements and developmental initiatives) that exist for test takers to engage in the testing tasks are virtually non-existent. Thus, testing during the emergency lockdown in academia has been, in the view of Wigdorowitz et al., especially challenging. That aside the authors provide a useful list to consider when undertaking online assessments.

Unlike the other articles in this series that provided more of a meta-view on conducting assessments, the last article in the series (Makhubela & Mashegoane, 2021) discusses the validation of the Fear of COVID-19 Scale. The sudden onset of the pandemic has meant that tools evaluating aspects of individuals physical and mental health in relation to the pandemic were a priority. Fear of COVID-19 is an everyday reality for the world’s population. In order to intervene on a large scale, this construct must be understood and amenable to comparison. Makhubela and Mashegaone found the psychometric properties of the scale to be sound in a sample of South African students. Furthermore, the results were comparable to what was found in other countries in and outside the African continent, suggesting that the instrument is useful for cross-national studies. The construct fear of COVID-19 is also argued to be distinct from other fears. Having a scale applicable across cultures bodes well for further conceptual developments and interventions in this area.

In conclusion, the articles in the series show that whilst the toll that the COVID-19 pandemic has exerted on many sectors of society in Africa, including industry and academia, is undeniable, there are positive outcomes. The outstanding positive outcomes for assessment is the transition and improved understanding of methods of training and the possible use of online assessment technologies equitably.
